# Mechanical Thrombectomy in Acute Terminal Internal Carotid Artery Occlusions Using a Large Manually Expandable Stentretriever (Tiger XL Device): Multicenter Initial Experience

**DOI:** 10.3390/jcm10173853

**Published:** 2021-08-27

**Authors:** Volker Maus, Sabeth Hüsken, Vladimir Kalousek, Grzegorz Marek Karwacki, Hannes Nordmeyer, Ilka Kleffner, Werner Weber, Sebastian Fischer

**Affiliations:** 1 Knappschaftskrankenhaus Bochum-Langendreer-Universitätsklinik, Institut für Diagnostische und Interventionelle Radiologie, Neuroradiologie, Nuklearmedizin, In der Schornau 23-25, 44892 Bochum, Germany; volker.maus@kk-bochum.de (V.M.); sabeth.huesken@kk-bochum.de (S.H.); werner.weber@kk-bochum.de (W.W.); 2 Subdivision of Interventional Neuroradiology, Department of Radiology, Clinical Hospital Center Sisters of Mercy, 10000 Zagreb, Croatia; vladozg@gmail.com; 3 Luzerner Kantonsspital, Diagnostische und Interventionelle Neuroradiologie, Radiologie und Nuklearmedizin Spitalstrasse, 6000 Luzern, Switzerland; grzegorz.karawacki@luks.ch; 4 Institut für Interventionelle Radiologie und Neuroradiologie, Neurozentrum Solingen, Radprax St. Lukas Hospital, 42697 Solingen, Germany; nordmeyer@gmx.com; 5 School of Medicine, Department of Health, Witten/Herdecke University, 58455 Witten, Germany; 6 Knappschaftskrankenhaus Bochum-Langendreer-Universitätsklinik, Klinik für Neurologie, In der Schornau 23-25, 44829 Bochum, Germany; Ilka.Kleffner@kk-bochum.de

**Keywords:** mechanical thrombectomy, acute stroke, Tigertriever XL, ICA terminus

## Abstract

Background: The recently introduced Tigertriever XL Device for treatment of cerebral vessel occlusions combines manual adjustability and maximum length in one device. In this study, we report our initial experience with the Tigertriever XL in terminal ICA occlusions. Methods: Retrospective multicenter analysis of acute terminal ICA occlusions treated by mechanical thrombectomy using the Tigertriever XL Device. Results: 23 patients were treated using the Tigetriever XL due to an acute occlusion of the terminal ICA. The overall successful reperfusion rate after a median of two maneuvers using the Tigertriever XL Device was 78.3% (mTICI 2b-3). In 43.5% (10/23) additional smaller devices were applied to treat remaining occlusions in downstream territories, which resulted in a final successful reperfusion rate of 95.7%. Device related complications did not occur. Two symptomatic intracerebral hemorrhages (sICH) were observed. Conclusions: The Tigertriever XL Device might be a helpful tool in the treatment of ICA terminus occlusions with large clot burden resulting in high reperfusion rates. This is mainly related to the manual adjustability of the device combined with the maximum length.

## 1. Introduction

Ischemic stroke due to an occlusion of the terminal internal carotid artery (ICA terminus) accounts for 20–30% of large vessel occlusions (LVO) within the anterior circulation [1]. Patients suffering from acute stroke caused by an ICA terminus occlusion have a poorer prognosis compared to LVOs in more distally located segments of the anterior circulation. Main reasons therefore are the larger clot burden with lower successful reperfusion rates associated with a higher incidence of embolism into downstream territories, while the impairment of the cerebral blood supply is greater due to mostly poor collaterals [2,3,4,5].

For LVOs within the anterior circulation, mechanical thrombectomy (MTE) using either a stent-retriever in combination with aspiration or aspiration alone is safe and effective [6,7]. With a rapidly growing number of devices and strategies for MTE the most promising concept to remove large clots (as in ICA terminus occlusions) remains unclear. Several studies indicated a higher first pass effect (FPE) and lower rates of embolism to a new territory (ENT) when stent-retrievers and in particular longer devices are used [8,9,10,11].

A recent study demonstrated higher successful reperfusion rates achieved with a manually expandable stent-retriever compared to conventional devices following a comparative analysis of MTE in acute LVOs within the anterior circulation [12]. The novel Tigertriever XL Device (Rapid Medical, Yoqneam, Israel) as the largest stent-retriever available to date is intended for the treatment of ICA terminus occlusions only. The design combines manual adjustability and maximum length in one device, as these properties might be crucial for successful reperfusion in LVOs due to large clots within the ICA terminus.

With this retrospective observational multicenter analysis of acute ICA terminus occlusions treated with the Tigertriever XL Device on an intention to treat approach we thought to evaluate our initial angiographic and clinical experience.

## 2. Materials and Methods

### 2.1. Data Analysis

We performed a retrospective analysis of the prospectively maintained stroke databases at the four participating neurovascular centers. All patients treated by MTE using the Tigertriever XL on an “intention to treat approach” due to an acute occlusion of the ICA terminus between July 2020 and June 2021 were included. An underlying proximal stenosis of the ipsilateral ICA treated by implantation of a stent prior to MTE (tandem lesion) was not an exclusion criterion in this analysis.

The diagnosis was confirmed by multimodal computed tomography (CT) including non-enhanced CT and CT-angiography, while CT-perfusion studies were not routinely performed. All pre-procedural non-enhanced CTs were retrospectively classified according to the Alberta Stroke Programme Early CT Score (ASPECTS). The clinical status of each patient was evaluated by the attending stroke neurologist according to the National Institutes of Health Stroke Scale (NIHSS) and the modified Rankin Scale (mRS) on admission and at discharge. A postprocedural non-enhanced CT was routinely performed within 24 h following the procedure. Patients that arrived within 4.5 h of symptom onset received intravenous thrombolysis (IVT), if eligible. The decision to perform MTE resulted from an interdisciplinary consensus of the neurological and neuro-interventional team members based on the 2015 guidelines of the American Heart and Stroke Association and the guidelines of the German Society of Neurology [7]. The final endovascular treatment strategy was left at the operator’s discretion, while only cases where the Tigertriever XL was used on an intention to treat approach were included.

The following data were analyzed in retrospect: successful reperfusion achieved using the Tigertriever XL Device only defined by the modified Thrombolysis in Cerebral Infarction scale score (mTICI) 2b and 3; the final mTICI (2b and 3) after additional thrombectomy maneuvers in downstream vascular territories were performed with suitable devices and the first pass effect (defined as mTICI 3) [13]. Further aspects evaluated included the number of MTE maneuvers with the Tigertriever XL up to a successful reperfusion and the evidence of subarachnoid hemorrhages (SAH) or symptomatic intracerebral hemorrhage (sICH) following the procedure.

The indication to use the Tigertriever XL is restricted to the treatment of ICA terminus occlusions due to its size. Therefore, additional occlusions in the downstream branches of the anterior or middle cerebral artery (whether preexistent or in the context of an ENT) were treated with different suitable devices.

### 2.2. Statistical Analysis

The statistical analysis of all variables was performed independently using Excel 2019 (Microsoft, Redmond, WA, USA) and IBM SPSS Statistics 27 (IBM, Armonk, NY, USA). Continuous variables are given as the median and range and independent variables are described as percentages.

### 2.3. Tigertriever XL Device/Endovascular Procedure

**Figure 1:**
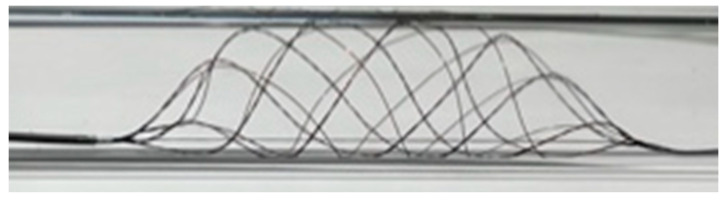
Expanded mesh of the Tigertriever XL Device.

The fundamental technical concept of the Tigertriever XL Device does not differ from the remaining Tigertriever Devices, whose design consists of a braided mesh of nitinol wires with the distal end connected to an inner wire (Figure 1). A control handle fixed to the proximal end of this inner wire enables the operator to stepwise open and close the mesh via the inner wire. This unique capacity proved to be effective for MTE in acute stroke due to LVOs [12,14]. The Tigertriever XL with its maximum length of 53 mm is expandable to a diameter ranging from 1.5 up to 9.0 mm, which results in a relevant shortening of the mesh. The device is compatible with a 0.021-inch microcatheter. At least in theory the Tigertriever XL is applicable in vessel diameters similar to the potential diameters of the device. We restricted the indication to use the Tigertriever XL to ICA terminus occlusions, especially with regard to the relevant length of the device assuming an increased likelihood of vessel injuries in further distally located smaller and curved arteries. This decision was in line with the initial advice of the manufacturer.

Procedures were performed under general anesthesia by experienced stroke interventionalists (at least five years of experience in endovascular stroke treatment) on different biplane angiographic systems (Siemens, Erlangen, Germany or Philips, Amsterdam, The Netherlands) at the contributing centers. After confirmation of the suspected ICA terminus occlusion by digital subtraction angiography a combination of an 8 and 6 French (F) guiding and aspiration catheter was placed within the target cervical ICA. Guiding and aspiration catheters were applied according to the institutional standard of the participating centers without a predefined technical setup. In cases of an accompanying cervical ICA stenosis reconstruction of the affected segment by balloon dilatation and a self-expandable stent was carried out prior or following the MTE procedure. The decision whether to perform the stent angioplasty before or after the MTE was left upon the operator’s decision. Placement of the Tigertriever XL Device does not differ from that of conventional stentretrievers, while the desired position was the terminal ICA with the distal end of the mesh reaching into the M1 segment. The device was then expanded stepwise until the mesh was assumed to cover the entire circumference of the ICA based on the information obtained from the previously performed CTA or a relevant deformation of the mesh occurred. The aspiration catheter was pushed upwards guided by the wire of the Tigertriever XL. Then, the device was gently pulled back under continuous aspiration. In the event of resistance recognized by the operator during retrieval the expansion of the mesh was reduced accordingly. Once a sufficient reperfusion (mTICI 2b-3) was obtained, the procedure was terminated. The maneuver was repeated in cases of a remaining occlusion of the ICA terminus using the Tigertriever XL or alternative devices, while the decision of which device to use was made by the operator.

In cases of successful reperfusion of the ICA terminus but a remaining or additional occlusion in the downstream arteries, the procedure was continued using standard devices appropriate for these downstream arteries of smaller caliber (MCA M1 or M2 and ACA A2). The decision whether to continue or to cease the procedure in these situations was made by the operator. However, the fundamental goal was a final mTICI 2b-3 result.

## 3. Results

Between July 2020 and June 2021, 23 patients (47.8% female, 52.2% male, median age 75, range 52–95 years) were treated using the Tigetriever XL Device due to an acute occlusion of the ICA terminus at the participating centers.

Time median intervals from symptom onset to reperfusion and from groin puncture to reperfusion were 223 min and 41 min (range 115–348 min and 13–137 min), while in 9 of the 23 cases (39.1%) the exact time of symptom onset remained ambiguous (wake-up stroke). In 8 of the 23 patients (34.8%), IVT was initiated prior to the endovascular procedure. In 26.1% (6/23) of all cases, an accompanying ipsilateral extracranial ICA stenosis was treated prior to the MTE procedure (*tandem lesion*).

**Figure 2:**
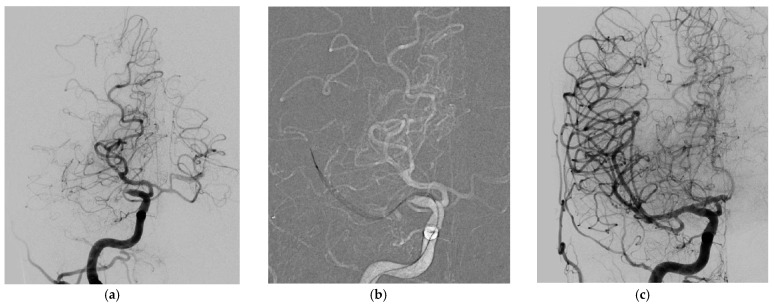
(**a**–**c**) ICA terminus occlusion treated successfully with one MTE maneuver using the Tigertriever XL Device. Acute occlusion of the ICA terminus distal to a fetal posterior cerebral artery origin, anterior-posterior view (**a**); Tigertriever XL Device placed within but mainly distal to the thrombus within the ICA terminus, anterior-posterior view (**b**); successful reperfusion after one maneuver (TICI 3), anterior–posterior view (**c**).

The median NIHSS and ASPECTS on admission was 16 (range 2–42) and 8 (range 5–10). The overall successful reperfusion rate (mTICI 2b-3) in the entire series was 95.7% (22/23 cases). The primary endpoint (successful reperfusion using the Tigertriever XL only) was accomplished in 18 of the 23 cases (78.3%) after a median of two attempts with the Tigertriever XL (range 1–3, Figure 2). The first pass effect (TICI 3 following a single maneuver) was 34.8% (8/23). In two procedures, the operator decided to switch to a different device after two and three unsuccessful maneuvers with the Tigertriever XL.

Additional devices used to treat occlusions in downstream smaller caliber arteries after successful reperfusion of the ICA terminus were used in 10 of the 23 cases (43.5%). An overview of the additional devices applied including the alteration of the TICI grades thereby is given in Table 1.

Technical failures of the Tigertriever XL Device or procedure-related complications as dissections or vessel ruptures did not occur. The postprocedural CT was without evidence of SAH in all of the cases. Two of the 23 patients experienced a sICH following the successful reperfusion observed on the postprocedural CT (8.7%). At discharge the median NIHSS was 11 (range 1–42) and the rate of mRS 0–2 was 26.1%. Five patients died related to the stroke but not the procedure itself (in hospital mortality 21.7%).

## 4. Discussion

With this retrospective analysis, we evaluated the safety and efficacy of MTE in ICA terminus occlusions using the Tigertriever XL Device. The overall successful reperfusion rate achieved with the Tigertriever XL alone and in combination with additional devices used for further occlusions in downstream arteries was 95.7%. This rate is slightly higher compared to those known from the randomized clinical trials (RCT) that were not restricted to ICA terminus occlusions but included LVOs within the anterior circulation in general, with successful reperfusion rates up to 86.0% [6].

ICA terminus occlusions are known for their poor clinical prognosis due to the large infarct volume with an unsatisfactory response to IVT, which is presumably related to a higher clot volume compared to more distally located occlusions within the Circle of Willis [15]. With the development and improvement of MTE techniques and devices successful reperfusion rates reported for terminal ICA occlusions improved continuously. While with IVT alone reperfusion rates were as low as 25%, the MERCI and Multi Merci trial in 2007 reported a successful reperfusion rate of 53% with the Merci Retriever alone and 63% with the Merci Retriever plus adjunctive endovascular treatments regarding intracranial ICA occlusions [16]. Since the RCTs that proved evidence for endovascular therapy in LVOs within the anterior circulation do not report subgroup analyses on specific locations regarding angiographic and clinical outcomes, exclusive data regarding MTE in the carotid terminus occlusions treated with contemporary stent-retrievers is sparse. In the largest single center series of 153 consecutive patients treated by MTE due to carotid artery occlusions within the first 4.5 h after symptom onset a TICI 2b-3 result was reported in 87.6%. Different to our series isolated occlusions of the extracranial internal carotid artery were included and MTE procedures were carried out using either a stent-retriever or thromboaspiration alone [17].

More than one-half of the cases included in our series were treated beyond the 4.5 h time window (12/23, 52.2%), which might potentially indicate cases of larger and fibrinogen richer clots. It is known that intracranial thrombus morphology and composition undergoes time-dependent changes, which include proximal and distal apposition as well as a progressive fibrin network propagation (*fibrin-rich white clot*) [18]. Fibrin-rich and longer clots are associated with increased reperfusion maneuvers, longer procedure time, less favorable clinical outcomes and reperfusion failures compared with red blood cell-rich clots following two recently published studies [19,20].

However, the median revascularization (symptom onset to reperfusion) and procedure time of 223 and 41 min found in our series is lower compared to the 317 and 71 min in the series described without cases in the late time window [17].

The lower overall procedure times found in our series might indicate the high efficacy of the Tigertriever XL Device. These findings are in line with those within the recently published Tiger Trial, with reduced procedure times using the Tigertriever in LVOs compared to results achieved with either Solitaire or Trevo devices. This potentially higher efficacy of the Tigertriever might be explained by the technical specifications of the device namely the manual adjustability of the radial force and with this the possibility to interact with the clot in combination with the larger size of the Tigertriever XL Device [12].

Interestingly, larger vessel diameters (>2 mm) were associated with a lower successful reperfusion following a subgroup analysis of the Tiger Trial that was carried out with standard versions of the Tigertriever without the Tigertriever XL, which might serve as a further argument for the value of device size in occlusions of larger vessels with higher clot burden [12].

Among ischemic strokes caused by LVOs ICA terminus occlusions are caused by longer thrombi with a larger thrombus volume as described above. Longer and larger thrombi are generally more difficult to retrieve, requiring more attempts and prolonging procedure time according to a study of Dutra et al., who analyzed 408 patients treated within the MR CLEAN Registry [21]. The radial force of a stent-retriever determines the interaction between the clot and the struts of the device. A higher radial force and larger gaps between the struts of a stent-retrieving device are associated with a more effective migration of the struts of the mesh into the thrombus, allowing for a successful and complete retrieval of the clot [14,22]. The stepwise manual expansion in the Tigertriever XL increases the radial force and with this the interaction with the clot, while the device can be adapted to the diameter of the ICA terminus or, if intended, its expansion can slightly exceed the diameter of the ICA. This is not possible in self-expandable stent-retrievers.

Longer stent-retrievers proved to be more efficient in an unselected group of LVOs treated with different stent-retrievers according to the study of Zaidat et al. [9]. In the subgroup of ICA terminus occlusions, the thrombus might extend from the MCA to any segment of the intracranial ICA. Here, a long stent-retriever offers the possibility to cover the occluded segment including its different diameters completely, which together with the adjustable amount of radial force, might explain the high efficacy of MTE in larger and longer thrombi using the Tigertriever XL.

The first pass effect of 34.8% found in our series is promising especially with regard to the subgroup of ICA terminus occlusions. Zaidat et al. performed a subgroup analysis of 354 acute ischemic stroke patients within the North American Solitaire Acute Stroke registry. They identified an overall first pass effect of 25.1%, while the first pass effect in the subgroup of ICA terminus occlusions was only 10.1%. The first pass effect was associated with improved angiographic, clinical, and safety outcomes, while ICA terminus occlusions were associated with lower rates of first pass effect [23].

A potential criticism of the strategy applied in this series might be the requirement of additional smaller devices to treat occlusions in downstream vessels since the Tigertriever XL Device is rather unsuitable for smaller caliber arteries. However, this *tailored* approach is in line with the increasing trend towards MTE in distal medium vessel occlusions (DMVO) [24].

Our study has several limitations that are mainly related to the retrospective design without fixed inclusion criteria whether to use the Tigertriever XL Device or alternative standard devices. Furthermore, the small sample size of this analysis does not allow for a reliable (matched pair) comparison of ICA terminus occlusions treated with different devices. Additional factors with a potential influence on the angiographic and clinical success of the procedure (e.g., impact of bridging IVT, operator experience) were not analyzed.

## 5. Conclusions

MTE of ICA terminus occlusions seems to be safe and effective using the Tigertriever XL Device. The technical properties together with the large size of the device allow for the successful removal of larger and longer clots. In some instances, additional smaller thrombectomy devices are helpful to treat remaining occlusions in downstream smaller arteries. Further prospective studies are needed to confirm the findings of this observational retrospective analysis.

## Figures and Tables

**Table 1:** Summarizes baseline clinical and angiographic results of all cases treated using the Tigertriever XL Device due to an acute occlusion of the ICA terminus.

Patient	Wake-Up 0 = N 1 = Y	IVT 0 = N 1 = Y	Tandem Occlusion	Symptom Onset to Reperfusion (Minutes)	Number of Passes with Tiger XL	Bail Out	mTICI	First Past Effect 0 = N 1 = Y	Additional Device	Final TICI	(Post)Procedural Complications 0 = N 1 = SAH 2 = sICH 3 = Dissection	NIHSS Admission (0–42)	NIHSS Discharge (0–42)	mRS Discharge (0–6)
1	1	0	0		1		3	1		3	0	12	5	2
2	0	1	0	246	2	pREset	0	0		2a	0	15	42	6
3	1	0	0		2		2a	0	3MAX	2b	0	19	42	6
4	0	0	0	278	3		3	1		3	0	18	8	3
5	0	0	0	115	1		3	1		3	0	20	5	2
6	0	1	0	223	2		2b	0	pREset	2c	0	9	7	3
7	0	1	0	348	2		2b	0	Tiger13	2c	0	20	14	4
8	0	0	0	218	2		3	0		3	0	15	14	4
9	1	0	0		2		3	0		3	0	17	11	3
10	0	0	0	175	1		3	1		3	0	12	1	1
11	0	0	0	304	1		3	1		3	0	14	3	1
12	0	1	0	223	1		3	1		3	0	23	8	4
13	0	1	0	151	1		3	1		3	0	12	1	0
14	0	1	0	219	2		2b	0	Solitaire	2c	0	16	18	5
15	0	1	0	244	2		2b	0	Tiger13	2c	0	26	16	5
16	1	0	1		1		2b	0	3MAX	2c	0	17	10	4
17	1	0	1		2		2a	0	Nimbus	2c	0	16	8	3
18	1	0	1		3	Nimbus	0	0	Aperio	2c	0	16	11	4
19	1	0	0		1		2a	0	Aperio	2b	0	21	42	6
20	0	0	0	270	2		2b	0	pREset	3	0	6	42	6
21	1	0	1		1		3	1		3	0	2	1	1
22	0	1	1	202	2		3	0		3	2	15	15	4
23	1	0	1		3		2b	0		2b	2	42	42	6
	Total N (%)	Total N (%)	Total N (%]	Median (Min)	Median	Total N (%)	2b-3 N (%)	Total N (%)	Total N (%)	2b-3 N (%)	Total N (%)	Median	Median	0–2 N (%)
	9 (9.1)	8 (4.8)	6 (6.1)	223	2	2 (8.7)	18 (78.3)	8 (34.8)	10 (43.5)	22 (95.7)	2 (8.7)	16	11	6 (26.1)

## Abbreviations

ICA terminus	terminal internal carotid artery
LVO	large vessel occlusion
MTE	mechanical thrombectomy
ENT	embolism into a new territory
IVT	intravenous thrombolysis
FPE	first pass effect
CT	computed tomography
NIHSS	National Institutes of Health Stroke Scale
mRS	modified Rankin Scale
SAH	subarachnoid hemorrhage
sICH	symptomatic intracerebral hemorrhage
F	French
RCT	randomized clinical trials
